# A Versatile Synthesis Approach and Interface Characterization of t‑ZnO@Metal Hydroxide/Oxide Heterostructures

**DOI:** 10.1021/acs.cgd.5c01604

**Published:** 2026-02-20

**Authors:** Barnika Chakraborty, Tim Tjardts, Berit Zeller-Plumhoff, Ulrich Schürmann, Anton Davydok, Dietmar Christian Florian Wieland, Haoyi Qiu, Alexander Reißmann, Nahomy Meling-Lizarde, Rajat Nagpal, Thomas Strunskus, Leonard Siebert, Rainer Adelung

**Affiliations:** † Chair for Functional Nanomaterials, Department for Materials Science, 9179 Kiel University, Kaiserstr. 2, Kiel 24143, Germany; ‡ Micro- and Nanosystems (MNS), KU Leuven − University of Leuven, Kasteelpark Arenberg 10, Leuven 3001, Belgium; § Chair for Multicomponent Materials, Department for Materials Science, 529440 Kiel University, Kaiserstr. 2, Kiel 24143, Germany; ∥ 28338 Institute of Metallic Biomaterials, Helmholtz-Zentrum Hereon, Max-Planck-Str. 1, Geesthacht 21502, Germany; ⊥ Data-Driven Analysis and Design of Materials, Faculty of Mechanical Engineering and Marine Technologies, University of Rostock, Rostock 18051, Germany; # Synthesis and Real Structure Group, Department for Materials Science, Kiel University, Kaiserstr. 2, Kiel 24143, Germany; ∇ Centre for Surface Chemistry and Catalysis, KU Leuven − University of Leuven, Celestijnenlaan 200F, Leuven B-3001, Belgium; 8 28338 Institute of Materials Physics, Helmholtz-Zentrum Hereon, Max-Planck-Str. 1, Geesthacht 21502, Germany

## Abstract

Functional ceramics play a key role in technology, particularly in piezoelectric sensors and actuators, ferroelectric power generation, and durable semiconductors used in sensors and memristors. In this study, we report a versatile wet chemical synthesis approach, converting the surface of functional tetrapodal zinc oxide (t-ZnO) to common metal hydroxides. We performed structural, morphological, and interface characterization and explored the subsequent application of various t-ZnO@metal hydroxide/oxide core–shell structures. The t-ZnO core was initially uniformly coated with different metal hydroxides, forming distinct platelets in a core–shell architecture. Interface studies were conducted to investigate the chemical, structural, and morphological properties of these hybrid microstructures using 2D scanning nano X-ray diffraction (XRD), scanning electron microscopy (SEM), transmission electron microscopy (TEM), bulk XRD, X-ray photoelectron spectroscopy (XPS), and Raman spectroscopy. Our findings highlight the potential of exceptional t-ZnO structures as versatile templates, offering their morphology for the synthesis of derived oxides and hydroxides of many other elements while leveraging their structural advantages.

## Graphical abstract

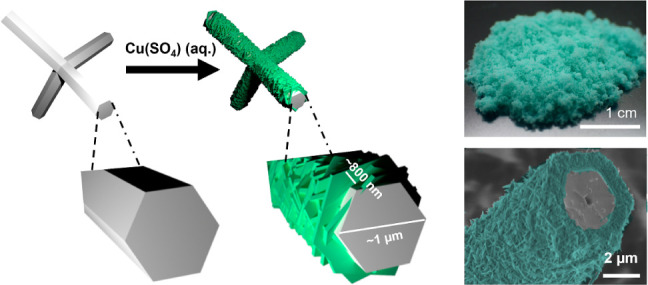

## Introduction

Metal oxide semiconductor-based materials have always remained at the center of attention for devices such as sensors and memristors.^1^ Often, these materials leverage unique properties, such as their high surface-to-volume ratio,^2^ tunable electronic properties,^2^ and sensitivity to surface interactions,^3^ to achieve enhanced sensitivity.^4^

Of various metal oxides, zinc oxide (ZnO) is a highly promising material for sensing applications,^5^ especially when engineered with intentional defects.^6^ ZnO possesses a wide direct bandgap (∼3.37 eV) and a large exciton binding energy (∼60 meV),^7^ making it intrinsically sensitive to UV light. However, the performance of ZnO-based sensors can be significantly enhanced by the presence of defects such as oxygen vacancies,^8^ zinc interstitials,^9^ and surface states.^10^ These defects act as charge trapping or recombination centers, which can modify the carrier dynamics upon illumination. For instance, oxygen vacancies can capture photogenerated electrons, increasing the lifetime of holes in the conduction band and thus enhancing the photoconductive response.^8^ Additionally, surface defects increase the adsorption and desorption activity of oxygen species, which alters the local charge distribution and enhances photocurrent generation.^11^ As a result, ZnO with controlled defect densities exhibits improved sensitivity, faster response and recovery times, and better stability in UV sensing.^12^ The tunability of its defect structure through methods like doping,^13^ annealing,^14^ or nanostructuring,^15^ factors which are also part of this study—further allows for optimization of sensor characteristics.

Zinc oxide (ZnO) composites with other transition-metal hydroxides/oxides have attracted significant attention in UV sensing applications, leveraging heterojunction formation, bandgap tuning, and enhanced charge separation. For instance, CuO–ZnO heterostructures have been shown to enable self-powered detection (365 nm) with robust photocurrent gain compared to pure ZnO nanorods.^16^ Co_3_O_4_–ZnO nanowire/rod heterojunctions have demonstrated visible-light photodetection, indicating that combining cobalt oxides with ZnO can extend detection into longer wavelengths.^17^ Though less explored for UV, analogous strategies involve Fe_2_O_3_–ZnO and NiO–ZnO systems as well.

In this work, we demonstrate a universal fabrication method for surface-specific reactions on t-ZnO metal oxide semiconducting (MOS) particles. This method is a wet-chemical step that reliably converts, due to its self-limiting parameters, the t-ZnO surface into nanostructures made from various hydroxide ceramics. The demonstrated hydroxide nanostructures include copper, cobalt, nickel, iron, and aluminum hydroxide as a shell with pristine t-ZnO as the core. A subsequent thermal oxidation can turn the hydroxides into oxides. The principle is depicted in Figure 1. The structures obtained by this approach are inexpensive and easy to fabricate. This can provide a platform for many desired MOS as a base, keeping the tetrapodal morphology intact and adding onto the properties from the surface. The single crystalline structure of the arms of t-ZnO is of particular significance. To reveal the core–shell structures of t-ZnO@metal hydroxide/oxide coatings in detail, 2D scanning nano X-ray diffraction (XRD) measurements of cross-sections are conducted for a better understanding of the interface and the thin layered composition of the shell.

**Figure 1:**
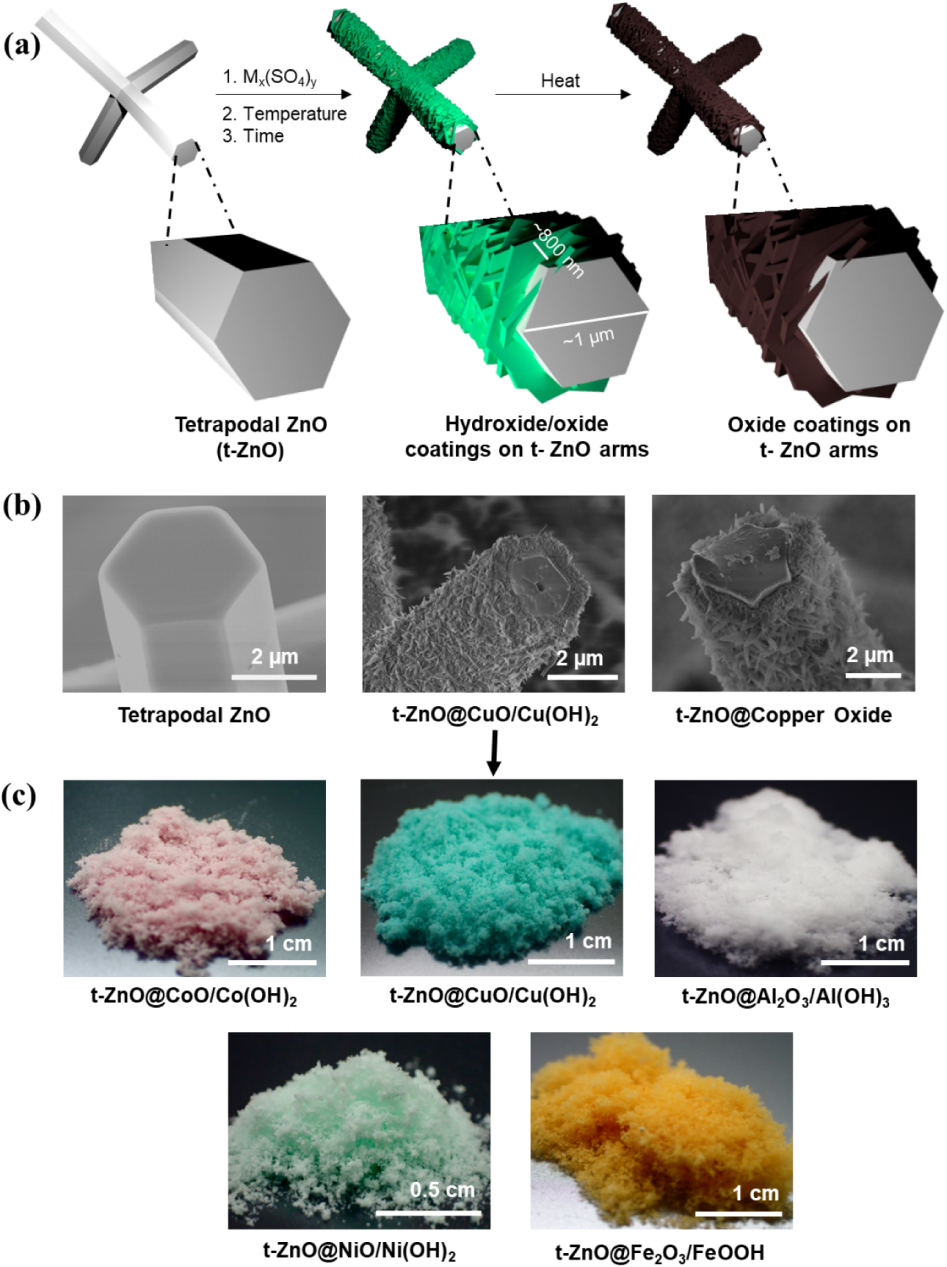
(a) Schematic representation of the synthesis of t-ZnO@metal hydroxides/oxides core–shell structures. (b) SEM images of t-ZnO, t-ZnO@CuO/Cu­(OH)_2_, and t-ZnO@Copper Oxide (Cu variations). (c) Images of powder samples of the metal derivatives coated t-ZnO.

## Materials and Methods

### Materials Used

Copper sulfate pentahydrate > 99% from Roth (Germany), cobalt sulfate heptahydrate > 99% from Merck, aluminum sulfate hydrate 98% from Merck (Germany), iron sulfate heptahydrate 99% from Merck, and nickel sulfate hexahydrate 98% from Roth (Germany). All chemicals were used without further purification.

### t-ZnO Synthesis

t-ZnO particles were synthesized using a flame transport synthesis method, following the procedures detailed in previous studies.^18^ The synthesis process involved the combination of zinc particles, sized <10 μm (Sigma-Aldrich, Germany), with polyvinyl butyral (PVB) powder (Kuraray Europe GmbH) at a weight ratio of 1:2. The mixture was heated inside an alumina crucible placed in a preheated oven set to 900 °C for about 30 min.

During the heating process, PVB reacts with the surrounding oxygen, shielding zinc from oxidation until it is evaporated. In the gaseous phase, the zinc atoms react with oxygen as soon as all of the PVB is consumed. The t-ZnO starts to nucleate and subsequently grows into tetrapodal-shaped microcrystals. After a period of 30 min, the crucible was carefully removed from the oven, and the resultant t-ZnO particles were collected as a distinctive white, fluffy powder.

### Coating of t-ZnO with Metal Hydroxides/Oxides

A concentration of 1 mg/mL of t-ZnO was added to a 0.1 M CuSO_4_ solution and maintained at room temperature for 1 h to obtain a layer of uniform thickness. Consequently, the resulting solution was carefully washed with water and subsequently dried in an oven at approximately 80 °C. The procedure is similar for the production of other metals, with a few notable exceptions. The concentration of t-ZnO was always maintained at 1 mg/mL. For Co and Ni, t-ZnO was added to 0.1 M CoSO_4_ and 0.1 M NiSO_4_ and kept at 80 °C for 2 and 24 h, respectively. For Al and Fe, 0.01 M Al_2_(SO_4_)_3_ and 0.01 M FeSO_4_ solutions were first cooled to 4 °C, followed by the addition of t-ZnO for 5 min and 1 h, respectively, at room temperature. The synthesized materials were further washed with water and dried in an 80 °C oven.

### XPS Sample Preparation

The XPS measurements were performed in an XPS UHV system (PREVAC Sp. z o. o.). The spectra were recorded applying a nonmonochromatic Al anode X-ray source at 300 W (15 kV, 20 mA). The base pressure inside the XPS analysis chamber was 1 × 10^–9^ mbar, which was provided by a scroll backing pump and a turbomolecular pump. Survey spectra were conducted in a binding energy range from 1300 to 0 eV with 3 iterations and a pass energy of 200 eV. Supporting Figure 1a shows these survey spectra, which were used to identify the binding energy regions of interest for the corresponding high-resolution spectra. The high-resolution spectra were measured within the binding energy regions of interest at 20 iterations and a pass energy of 50 eV. The evaluation of the data was performed in Casa XPS (version 2.3.23). The spectra were charge-corrected by setting the main C 1s peak at a binding energy of 284.8 eV. For the acquired high-resolution data, Gaussian–Lorentzian curves were utilized as fitting functions. From the positions of these fitting functions, the corresponding peak positions were approximated.

### Raman Characterization

To investigate the composition of chemical constituents of the shell layer on the t-ZnO structures, chemical analysis of the prepared samples was performed using micro-Raman spectroscopy under ambient conditions. The measurements were carried out using a WITec alpha 300 RA system (WITec GmbH, Ulm, Germany), which is equipped with a triple-grating spectrometer and a CCD detector. The grating parameters were set at 600 g/mm with a wavelength of 500 nm. A green laser with a wavelength of 532 nm served as the excitation source. Before conducting the investigations, the spectrometer was calibrated by using a Si wafer.

### XRD Sample Preparation

The materials underwent primary structural characterization using Rigaku SmartlabXRD at 45 kV and 200 mA, employing Cu K_α1_ radiation (λ = 1.54 Å) within a 2θ range of 20–80°. To minimize interference, the measurements were conducted on a nonmetallic holder. The obtained signals provided evidence of the crystalline nature of the samples.

### Interface Characterization Sample Preparation

We developed a methodology for preparing cross-sections of tetrapods for two-dimensional nano XRD studies. The tetrapods were first broken down into their arms as ZnO microrods by sonicating them in ethanol overnight. These rods, once obtained were consequently mixed with ethanol and PVB in a ratio of 1:3:1 (by mass), forming a viscous paste. This paste was then extruded through a nozzle with an opening diameter of 0.84 mm and allowed to dry. This led to the formation of polymer rods with a diameter of around 5 mm, in which the tetrapod arms were aligned in the direction of extrusion. These structures were cut with an ultramicrotome (Reichert Ultracut S) that cuts both the polymer and the embedded tetrapod microrods into these slices of roughly 1 μm diameter. By previously aligning the rods within the PVB, the probability of receiving 2D cross-sections of the rods was increased. The sliced PVB, including the t-ZnO cross-sections was then deposited onto TEM grids. The process is schematically depicted in Figure 2.

**Figure 2:**
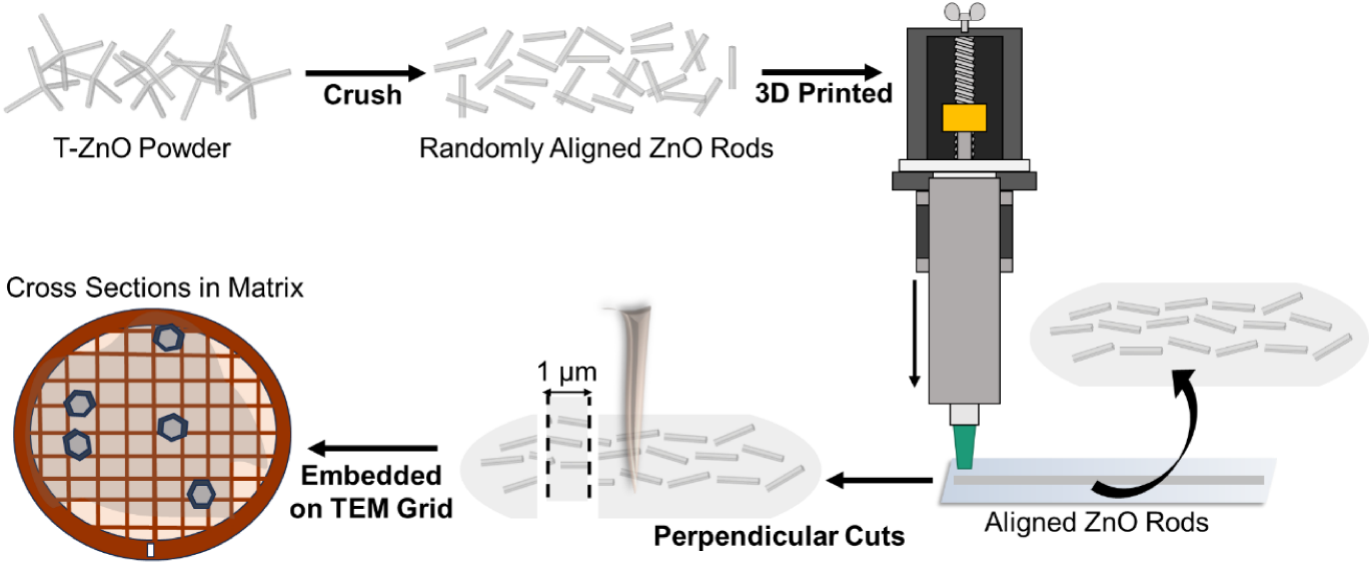
Schematics of sample preparation for 2D nanodiffraction.

### 2D Scanning Nano XRD

The TEM grids were clamped into a custom holder for the 2D nano XRD studies. 2D scanning XRD was conducted with a beam size of 250 nm × 350 nm at an energy of 19.75 keV. The measurements were conducted at the nanodiffraction end-station of the P03 beamline operated by Helmholtz-Zentrum Hereon at PETRA III (Deutsches Elektronensynchrotron, Hamburg, Germany). For XRD measurements, an Eiger 9 M detector (DECTRIS Ltd., Baden-Daettwil, Switzerland) was placed behind the sample at a distance of 0.223 m, and additionally, an X-ray fluorescence (XRF) detector (SSD 123, AMPTEK Inc., Bedford, MA, USA) was placed at a distance of 3 cm to record the elemental information. Both measurements were done in parallel using an exposure time of 15 s. The calibration of the XRD signal was performed using lanthanum hexaboride as the calibrant, while the XRF signal was calibrated using Cu and Ti sheets of 7.5 μm thickness, respectively. Following data calibration, reduction, and solid angle and polarization correction were done with pyFAI.^19^ The data were evaluated in terms of elemental maps, XRD peak position, and width using in-house scripts in Matlab (R2024b, The MathWorks, Inc., Natick, US), as previously described.^20^

### Electron Microscopy

The morphology and chemical composition were analyzed using scanning electron microscopy with a ZEISS Gemini REM system operating at 4 kV and 10 μA. The powdered sample was directly attached in small quantities by using carbon tape on the SEM stage for measurements. For further analysis, transmission electron microscopy (TEM) was carried out using an FEI Tecnai F30 G^2^ microscope. TEM samples were prepared by grinding the material and placing a small amount on a Ni grid, avoiding Cu grids due to the presence of CuO in the sample. The chemical composition was further proven by energy dispersion spectroscopy (EDS) from Oxford Instruments Ultim Max 65 at the SEM and with an EDAX (Si/Li) detector at the TEM.

## Results and Discussion

### Morphological Characterization

Morphological studies reveal core@shell structures of the semiconductor, t-ZnO, in the core as the sensing material, with a shell layer of variable metal hydroxide/oxide as shells. The composition of the shells were marked in the SEM images and derived from some of the other investigations. Figure 1 thus showcases the core@shell structures using the exact coating colors from the photograph (e.g., pink for Co) to provide clear visual guidance. The approach remarkably proved to be self-limiting. This leads to the hypothesis that the growth becomes diffusion-limited, i.e., the transport of educts or products to or from the interface is the bottleneck for the reaction. This then leads to the hypothesis that the coatings are dense at their core, not allowing for the direct contact of the medium and the zinc oxide interface.

Additionally, it can be observed that the tips of each tetrapodal particle remain pristine and do not react. This lends to the hypothesis that nucleation is dependent on the crystal face. Since t-ZnO particles are single crystals, the tips of each arm represent (0001) facets, while the sides are (010–1) facets. The facets on the sides also possess steps and kinks because the zinc oxide tetrapods are tapered, which leads to edge defects and exposed bonds that initiate nucleation.^21^ The (0001) facets are either oxygen- or zinc-terminated and are commonly the most stable facets in t-ZnO. Therefore, one only observes the growth of hydroxide platelets on the sides.

The approach is versatile and reproducible for a range of metals, from s-block aluminium (Al) to transition d-block elements, namely, copper (Cu), cobalt (Co), iron (Fe), and nickel (Ni). The coated layer thickness can be controlled and varied as per experimental and applicative requirements with the variation of concentration, reaction time, and experimental temperature conditions, as shown in Supporting Figure 1. For Cu, Co, and Ni, the growth of platelets is observed on the surface of t-ZnO. Among these, growth is slowest for Ni, followed by Co, and then Cu. The self-organized patterns of platelet formation, often appearing at a fixed distance, may be explained by the following hypothesis: the reaction likely involves a two-step process. In the first step, an intermediate species is formed in solution that diffuses a certain distance before being deposited through a second reaction. When the intermediates are formed at point defects, the intermediate concentration profile is spherical around the point defect. If there are multiple such sources for intermediates, then the overlap between their concentration profiles forms a plane. In these planes, the deposition manifests as platelets. The platelet’s orientation is likely determined by preferential growth along planes with minimal steric hindrance. For instance, the stepwise growth of t-ZnO could result in shell layers orienting along specific planes.

In contrast, the structures formed on Al and Fe differ significantly. Growth for these materials seems to occur much faster, making it challenging to control the reaction and achieve a defined thickness. To slow the reaction, Al and Fe solutions were cooled prior to the addition of t-ZnO. Despite this, the reaction proceeds much faster compared with Cu, Co, and Ni. Specifically, Al reacts in just 5 min and Fe in 20 min in cold solutions to achieve a thickness of 100–200 nm, whereas Cu requires 1 h at room temperature, and Co and Ni take 2 and 24 h, respectively, at an elevated temperature of 80 °C. The latter three can reach up to 800 nm in this time period.

The rapid reaction of Al could be attributed to much higher rate constants for the deposition of the products or a one-step reaction rather than a two-step reaction. Both would lead to the immediate deposition at the site of generation rather than the diffusion of intermediates, resulting in random ball-like growth rather than organized platelets. In the case of Fe, the structure exhibits a mix of both ball-like and template-like formations. Elements like Cu, Co, Fe, and Ni are reactive in their +2 oxidation states.^22^ In contrast, Al in the +3 oxidation state is the only known stable state for Al oxides and hydroxides, resulting in an initial formation in that oxidation state. Fe forms a mixture of +2 and +3 oxidation states and exhibits an intermediate reaction time, supporting this hypothesis. The purpose of this study was to understand the mechanism behind the versatile yet simple approach of wet chemical synthesis toward a platform based on different metals, keeping the tetrapodal structure intact for several applications, including UV sensing. The mapping of t-ZnO@CoO/Co­(OH)_2_ depicts the exact location of the existence of the ZnO and Co shell. However, we also find sulfur from the precursor sulfates (except for Fe). The additional maps of the other core@shell structures (except Co) are enlisted in Supporting Figure 2a,b,c.

Beyond providing morphological information, the microscopic observations also support the compositional uniformity of the shell layers. SEM and TEM images reveal continuous coverage of the side facets of the t-ZnO arms by metal hydroxide/oxide platelets, with no extended uncoated regions observed along the arm length. Although the shell exhibits a platelet-like morphology rather than a perfectly smooth film, the homogeneous spatial distribution of these platelets indicates uniform chemical conversion of the t-ZnO surface. This behavior is consistent with a surface-limited reaction mechanism, in which nucleation occurs across equivalent crystallographic facets, leading to compositionally uniform shell formation despite local morphological roughness.

### Structural Characterization

The structural characterization was performed with XPS analysis, followed by Raman and XRD. XPS analysis was performed to investigate the chemical state of the t-ZnO@metal hydroxide/oxide surfaces. Figure 4a–e shows the high-resolution metal 2p core-level spectra with fitting functions, while Figure 4f presents the fitted high-resolution Zn 2p core-level spectrum of the t-ZnO@CuO/Cu­(OH)_2_ sample. Moreover, the O 1s peak was analyzed with fitting functions to complement the evaluation of the metal 2p spectra. Figure 5a–e shows the corresponding O 1s spectra. Overall, the combined analysis of metal and O 1s peaks provides a basis for interpreting the chemical species present on the surface of the t-ZnO@metal hydroxide/oxide samples. As a small review, Figure 5f presents a summary table of the XPS analysis outcomes, highlighting the compounds that are most likely present on the surfaces of the samples.

**Figure 3:**
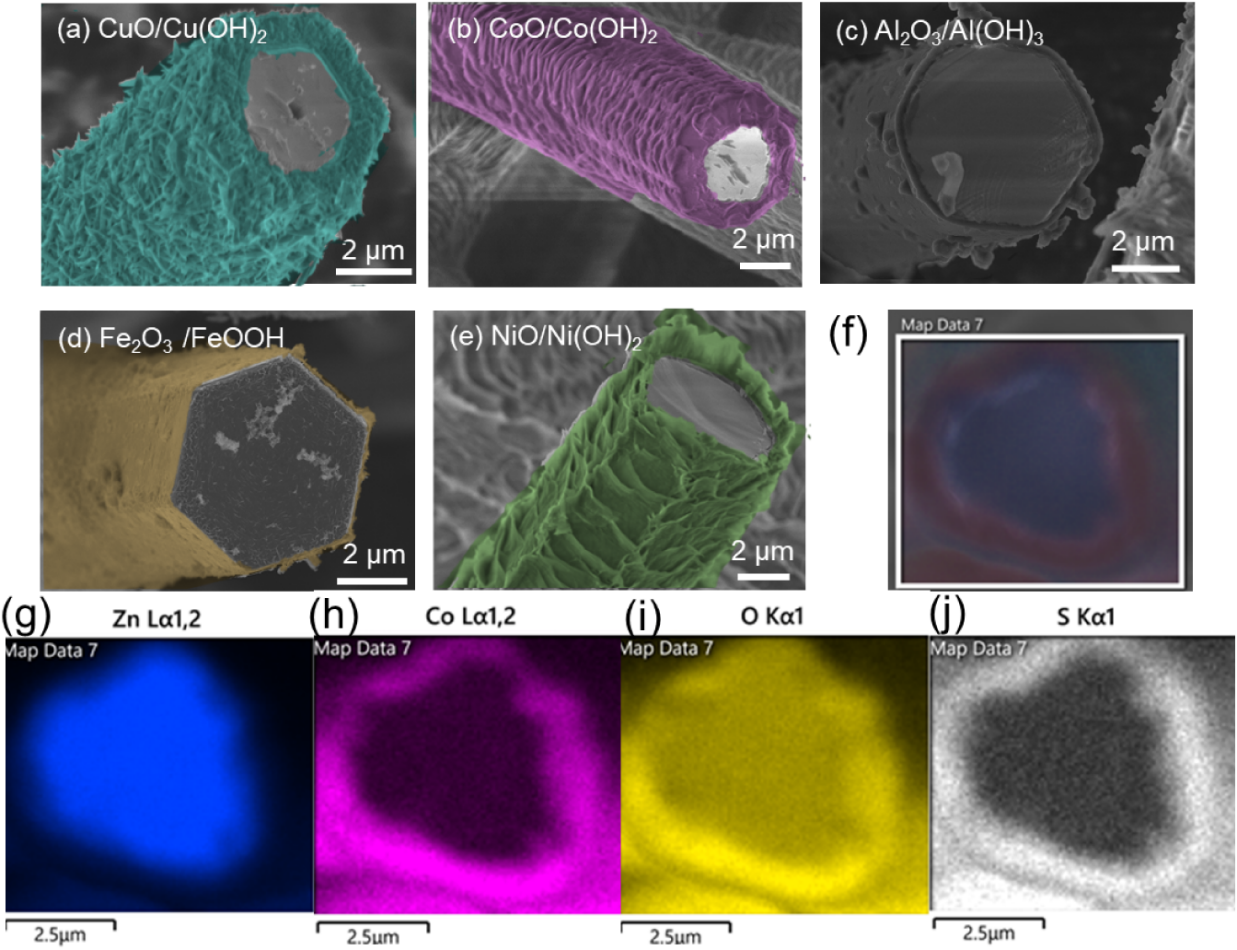
SEM images of (a) t-ZnO@CuO/Cu­(OH)_2_, (b) t-ZnO@CoO/Co­(OH)_2_, (c) t-ZnO@Al_2_O_3_/Al­(OH)_3_, (d) t-ZnO@Fe_2_O_3_/FeOOH, and (e) t-ZnO@NiO/Ni­(OH)_2_, with the shell layer colored according to the corresponding real colors of the samples while mapping. (f–j) Electron images and SEM–EDX maps of corresponding elements for t-ZnO@CoO/Co­(OH)_2_.

**Figure 4:**
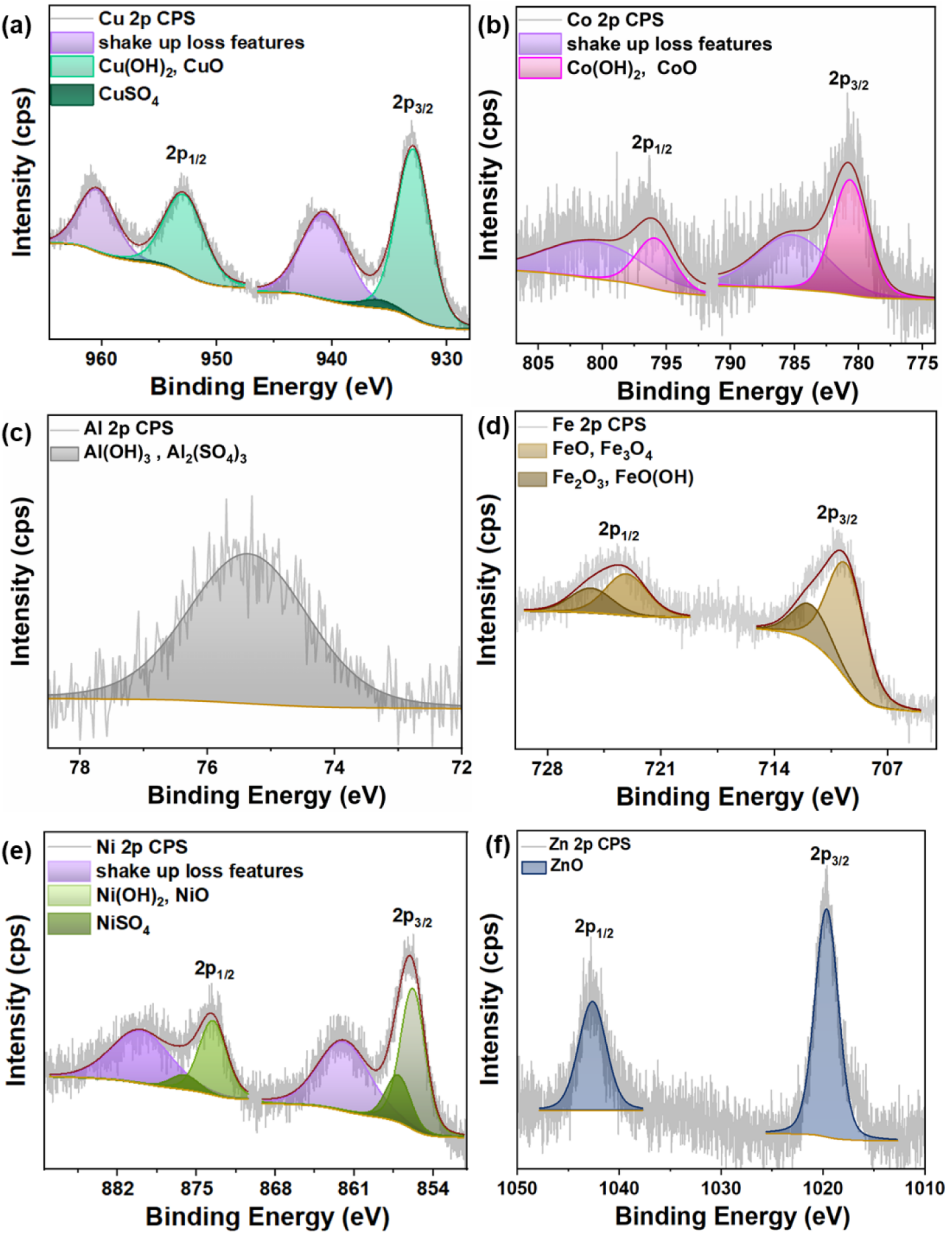
(a–e) XPS Cu 2p, Co 2p, Al 2p, Fe 2p, Ni 2p core-level spectra for the respective metal hydroxide/oxide shells, and (f) Zn 2p spectrum of the t-ZnO@CuO/Cu­(OH)_2_ sample representing t-ZnO from the core of the core–shell t-ZnO@metal hydroxide/oxide structures.

Figure 4a shows the Cu 2p core-level spectrum. It contains two main segments characteristic of the spin–orbit splitting of Cu 2p. At higher binding energies, the 2p_1/2_ segment prevails, and at lower binding energies, the 2p_3/2_ segment is present.^23,24^ Each of these segments contains three fitting functions. The highest binding energy fitting function most likely associates with shakeup processes upon photoemission.^25^ Such pronounced shakeup features strongly suggest the presence of Cu­(II) species on the sample surface.^24–27^ On the other hand, the appearance of pronounced shakeup features also suggests that metallic Cu and Cu­(I) species are less likely.^23^ The intermediate binding energy component is 936.0 eV in the Cu 2p_3/2_ segment. This binding energy position matches closely with the reported binding energy position of 935.5 eV for CuSO_4_.^28,29^ ^,^ However, this component has a comparably small relative intensity. Despite this, a significant S 2p peak was detected, as shown in Figure 3ba. The S 2p peak has an approximate binding energy position of 169.2 eV, which also matches with the reported binding energy of 168.8 eV for CuSO_4_.^29^ Therefore, CuSO_4_ is most likely part of the sample surface, and its fit as a possible CuSO_4_ component in the Cu 2p spectrum is reasonable. The lowest binding energy fitting function has a binding energy position of approximately 932.9 eV in the Cu 2p_3/2_ segment. This is very close to reported values of 933.8 and 933.45 eV for Cu­(OH)_2_ ^26^ and CuO^27^ respectively. The shape of the shakeup features, which lack a noticeable multiplet in the Cu 2p_3/2_ segment, indicates the dominance of Cu­(OH)_2_ over CuO at the sample surface.^26,27^ However, here, a lack of energy resolution could be another possible reason for the absence of distinguishable multiplets in the Cu 2p_3/2_ shakeup feature. Therefore, to make a more refined distinction between possible O-including components, the O 1s spectrum from Figure 5a must be evaluated. Here, the corresponding spectrum contains three fitting components. The lowest binding energy component at approximately 529.4 eV matches exactly with the reported value for lattice oxygen in CuO.^27^ Moreover, it is very close to the reported value of 530 eV for ZnO nanorods.^30^ A possible presence of t-ZnO at the sample surface is likely due to the presence of a significant Zn signal in the survey spectrum shown in Supporting Figure 3a as well as the lack of complete coating at the top of the t-ZnO tetrapod arms, as seen in the SEM images of Figures 1 and 3. The main O 1s component is located at intermediate binding energies of approximately 531.0 eV, which matches well with the reported value of 530.65 eV^26^ for Cu­(OH)_2_. Its high relative intensity strongly indicates the dominance of Cu­(OH)_2_ over other CuO and CuSO_4_ at the sample surface. The highest binding energy fitting function at approximately 532.1 eV associates well with the reported value of 532.0 eV for CuSO_4_.^29^ On the other hand, this position also aligns with possible adsorbed oxidized hydrocarbons^31^ and ZnSO_4_.^32^ The hydrocarbons are very likely since the sample was exposed to the atmosphere and prepared in a wet chemical process. In fact, similar arguments for hydrocarbons hold for each sample, judging from the O 1s peaks in Figure 5. The latter possible ZnSO_4_ component is very unlikely due to the strong presence of t-ZnO in the template shown by X-ray diffraction in Figure 7.

The spectrum in Figure 4b presents the spin–orbit splitting of the Co 2p core level. The 2p_1/2_ region is at higher binding energies, while the 2p_3/2_ region is prominent at lower binding energies.^33,34^ Each region contains two distinct fitting functions. The fitting function at the higher binding energy is typically attributed to shakeup losses occurring during photoemission.^35^ These shakeup features observed for both spin–orbit split regions indicate strong evidence for Co­(II) presence on the surface, rather than metallic Co or Co­(III). Here, studies demonstrated that metallic Co and Co­(III) oxides show, in contrast to Co­(II), an absence of shakeup losses in the 2p_3/2_ region.^36,37^ The binding energy of the main peak in the Co 2p_3/2_ segment is at approximately 779.8 eV, which does not correspond to the values between 782.2 and 781.0 eV reported in the literature for Co­(OH)_2._ ^38,39^ On the other hand, binding energy values for CoO are reported to be between 781.2 and 780.0 eV.^40,41^ At first glance, this indicates that the CoO compound is dominant over Co­(OH)_2_. However, the recorded data do not allow for the exact distinction between both chemical species because a second fit was not reasonably possible due to the relatively high noise and energy overlap of the reported CoO and Co­(OH)_2_ regions. Thus, the main fit of Co 2p_3/2_ represents both possible CoO and Co­(OH)_2_ species. For a distinction between these components, the corresponding O 1s spectrum in Figure 4b must be analyzed. Here, the spectrum of the O 1s shows three fitting functions. The component with the lowest binding energy is at approximately 529.3 eV and can be attributed to the lattice oxygen in t-ZnO.^42^ This finding is further supported by the prominent Zn 2p peak identified in the survey spectrum shown in (Supporting Figure 3ab). Nevertheless, the lattice oxygen in CoO should not be excluded, given the referenced value of 529.7 eV.^43^ However, a possible CoO component is most likely present in smaller quantities compared to Co­(OH)_2_ due to the low relative intensity of the corresponding fit. The highest binding energy component was identified at approximately 532.3 eV, which suggests the presence of CoSO_4_ on the sample surface. The S 2p spectrum shown in (Supporting Figure 3ab) supports this interpretation. The S 2p peak position is at approximately 169.1 eV and indicates CoSO_4_ as it is reported at 168.2 eV in the S 2p spectrum.^44^ The dominant binding component is at approximately 530.9 eV, which matches with the reported value for Co­(OH)_2_.^45^ Its high relative intensity suggests a predominant presence of Co­(OH)_2_ over CoO and CoSO_4_ on the sample surface.

Figure 4c exhibits the Al 2p core-level spectrum. The spectrum contains one main peak at approximately 75.4 eV. As a comparison with the literature, the Al 2p peak value for Al­(OH)_3_ has been reported at around 74.5 eV^46^ while Al_2_O_3_ has been reported between 76.2 and 73.0 eV in the Al 2p regime, according to the NIST database.^47^ Thus, the extracted Al 2p binding energy position of 75.4 eV suggests the presence of Al­(III) on the sample surface.^48^ Here, the nanostructured nature of the sample may contribute to slight chemical shifts in binding energy.^49^ Regarding a possible Al_2_O_3_ contribution, Tago et al. reported a binding energy of 73.4 eV for a clean surface of an Al_2_O_3_ single crystal.^50^ Due to the relatively high difference from the extracted value of 75.4 eV, this study gives a small indication that an Al_2_O_3_ component is unlikely to be dominant at the sample surface. Nevertheless, a detailed analysis of the O 1s spectrum from Figure 5c is necessary to distinguish between the possible Al­(III) species associated with oxygen. Figure 5c displays the O 1s spectrum with three defined fitting components. The lowest binding energy component is at approximately 530.5 eV and corresponds to the reported value for lattice oxygen in t-ZnO.^30^ The survey spectrum in (Figure S3ac) reveals a significant Zn signal, further suggesting the possible presence of t-ZnO at the sample surface, most likely due to an incomplete coating at the ZnO tetrapod arms. Additionally, a binding energy of 530.7 eV has been reported for Al_2_O_3_ in an Al_2_O_3_/ZnO thin-film composite.^51^ This also aligns with the extracted position of 530.5 eV for the lowest binding energy component and indicates that Al_2_O_3_ might be present as a minor contribution on the sample surface. On the other hand, the main O 1s peak is located at approximately 531.9 eV, indicating the hydroxides. Considering that this component shows the highest relative intensity in the O 1s peak, Al­(OH)_3_ is most likely the predominant species on the sample surface. The higher binding energy component, located at approximately 532.6 eV, is attributed to Al_2_(SO_4_)_3_, as it closely aligns with the reported value of 532.5 eV. Moreover, the S 2p peak in (Supporting Figure 3bc) approximately 169.9 eV, aligns very well with the reported value for Al_2_(SO_4_)_3_ at the same binding energy.^52^ Therefore, a minor amount of Al_2_(SO_4_)_3_ at the sample surface is plausible. The reported Al 2p signal of Al_2_(SO_4_)_3_ at 74.2 eV^52^ does not exactly match with the extracted Al 2p peak position of 75.4 eV from Figure 4c. However, the indications of O 1s and S 2p and the overall small contribution still justify a possible minor Al_2_(SO_4_)_3_ presence.

**Figure 5:**
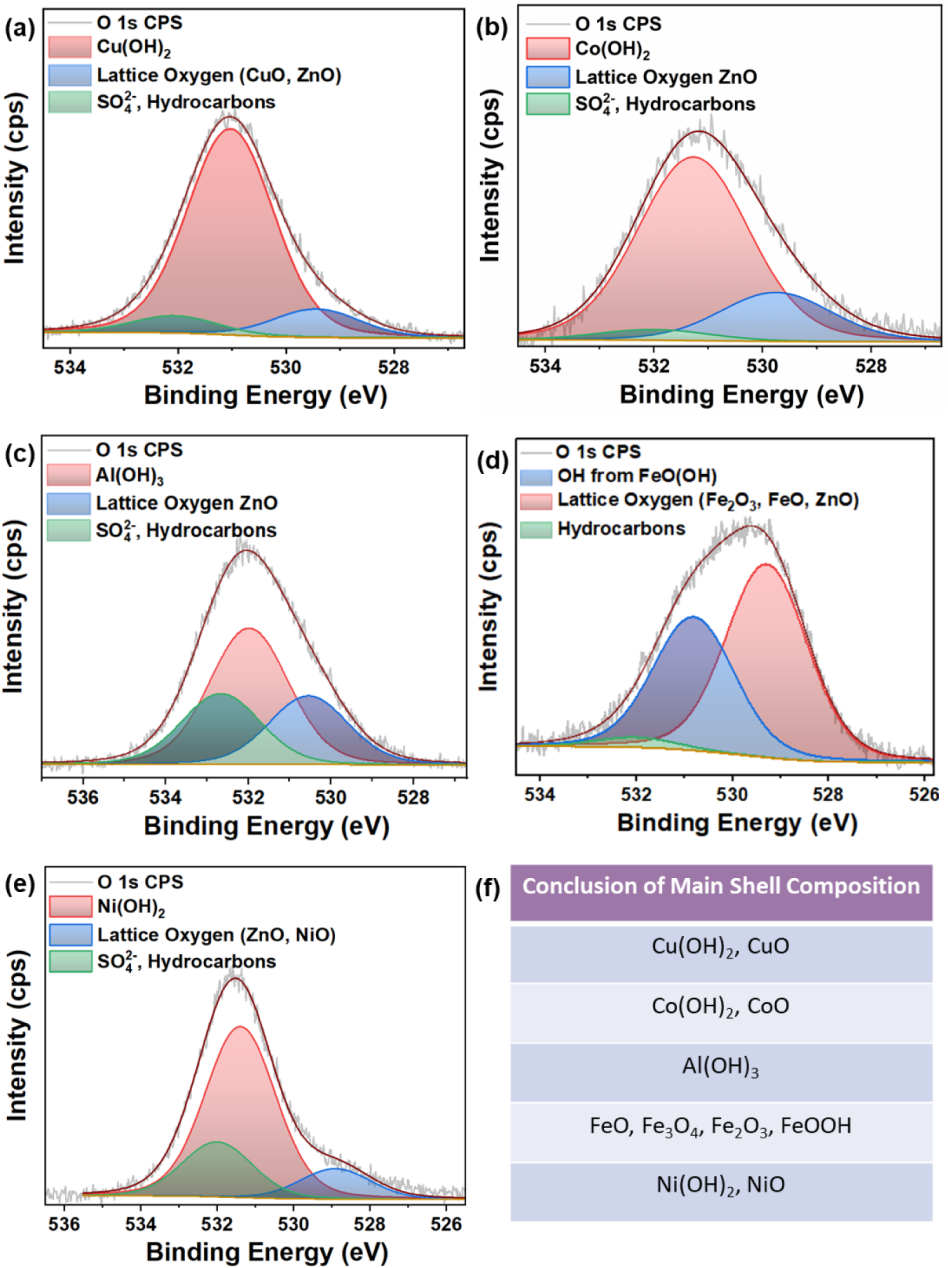
(a–e) O 1s XPS analysis for respective t-ZnO@metal hydroxide/oxide core–shell structures, and (f) summary table for the main shell composition of all core@shell structures.

Figure 4d presents the Fe 2p core-level spectrum, which exhibits two primary regions corresponding to the 2p_1/2_ and 2p_3/2_ spin–orbit splitting. Each segment consists of two distinct fitting functions. The highest binding energy fitting function for the 2p_3/2_ splitting is located at approximately 711.7 eV. This indicates the presence of Fe­(III) on the sample surface, as it is reported to have a value around 711.0 eV.^53^ In contrast, the lower binding energy primary Fe 2p_3/2_ component, located at approximately 709.5 eV, suggests the presence of Fe­(II) species, as the NIST database reports values ranging from 708.3 to 710.8 eV.^54,55^ However, since the samples were prepared in a nonreducing atmosphere, the formation of Fe­(II) components such as FeO and Fe_3_O_4_ is unlikely. These conditions favor the formation of Fe­(III) species over Fe­(II), making the presence of FeO and Fe_3_O_4_ less probable. Nevertheless, the lower energy components in the Fe 2p state suggest the presence of Fe­(II) species at first glance. Therefore, further analysis of the O 1s spectrum shown in Figure 5d is required to distinguish between the Fe oxide components, providing additional insights into the oxygen binding environments on the sample surface. Here, the spectrum contains three fitting components. The lowest binding energy component at approximately 529.3 eV is likely attributed to lattice oxygen in t-ZnO,^56^ supported by the significant Zn 2p peak, as shown in (Supporting Figure 3ad). Additionally, this peak may also be attributed to lattice oxygen in Fe_2_O_3_ as it aligns closely with the reported value of 529.7 eV.^57^ Furthermore, the presence of lattice oxygen in the Fe_3_O_4_ component cannot be excluded, considering the reported value of 529.5 eV.^58^ The intermediate peak at approximately 530.8 eV can be attributed to the presence of FeO­(OH) in accordance with the reported value of 531.2 eV.^59^ The highest binding energy component is primarily attributed to hydrocarbons, as no S 2p signal was detected in this sample. In sulfate solutions, Fe­(II) oxidizes to the more stable Fe­(III) upon hydroxide formation, driving rapid and complete conversion without residual reactants. In contrast, Co, Ni, and Cu remain in the II oxidation state throughout the process, lacking a similar stability transformation. Consequently, their nucleation rates are comparatively slower, often resulting in the retention of sulfur from sulfate precursors.

The Ni 2p core-level spectrum presented in Figure 4e displays two primary regions, indicative of 2p_1/2_ and 2p_3/2_ spin–orbit splitting. Each of these segments contains three distinct fitting functions. The component with the highest binding energy associates to shakeup features of Ni­(II) species.^60^ Here, the pronounced shakeup satellites strongly indicate that the presence of NiS, NiS_2_ and Ni_3_S_4_ is improbable.^61^ The intermediate fitting function, located at approximately 857.0 eV in the Ni 2p_3/2_ segment, aligns precisely with the reported value for NiSO_4_.^62^

Despite the relatively low intensity of this component, a notable S 2p peak is evident from (Supporting Figure 3bd). This peak, observed at approximately 168.9 eV, matches closely with the reported value of 169.4 eV for NiSO_4_.^32^ The main peak in the Ni 2p_3/2_ region is located at approximately 855.7 eV, which agrees with the reported 855.1 eV for Ni­(OH)_2._ ^63^ Moreover, NiO has been described to exhibit a binding energy around 854.0 eV,^64,65^ making its presence on the sample surface less likely at first glance. However, NiO also shows a distinct double peak feature in its main Ni 2p_3/2_ component, including binding energy positions greater than 854.0 eV.^64,66^ Therefore, the extracted Ni 2p_3/2_ position at approximately 855.7 eV cannot completely exclude the presence of NiO. Consequently, the O 1s spectrum must be considered to further distinguish the different possible O-containing Ni species. Figure 5e displays the corresponding O 1s spectrum with three fitting functions. Here, the lowest binding energy subpeak at approximately 528.9 eV associates with lattice oxygen in t-ZnO.^56^ Nevertheless, NiO is reported to be at approximately 529.1 eV.^67^ This suggests the presence of both t-ZnO and NiO in the sample. However, their quantity is relatively low, as indicated by the low relative intensity of the component at lower binding energy, which is in agreement with the preceding analysis on the Ni 2p spectrum in Figure 4e. At higher binding energies, a peak appears at approximately 532.0 eV, closely corresponding to the reported value of 532.1 eV for NiSO_4._ ^68^ A minor contribution of NiSO_4_ is also supported by the S 2p signal shown in (Supporting Figure 3bd). Here, the extracted peak position of 168.9 eV strongly matches with the reported value of 169.1 eV for NiSO_4._ ^67^ The main O 1s peak is at approximately 531.3 eV, which can be attributed to Ni­(OH)_2_ present on the sample surface, as it aligns with the value of 530.6 eV reported for Ni­(OH)_2_ in the literature.^69^

### Raman Characterization

For t-ZnO, the majority of the peaks match with the literature. We see the E_2L_, 2E_2L_, E_2H_-E_2L_, A_1_(TO), E_1_(TO), E_2_, E_2H_+E_2L_, A_1_(LO), E_1_(LO), and 2.E_2H_+E_2L_, 2A_1_(LO) peaks at 101, 204, 332, 387, 414, 439, 538, 585, 655, 1100, and 1144 cm^–1^, respectively. The hexagonal ZnO crystal adopts a wurtzite structure with space group C_6_v^4^ (P63mc), containing two formula units per primitive cell. This structure gives rise to six Raman-active phonon modes at 101 cm^–1^ $\left(E_{low}^{2}\right)$ 381 cm^–1^ (A_1_ TO), 407 cm^–1^ (E_1_ TO), 437 cm^–1^ (high E_2_), and 583 cm^–1^ (E_1_ LO) in the first-order Raman spectrum. In a backscattering configuration with incident light normal to the sample surface, the Raman selection rules predict that only the E_2_ and A_1_ LO modes should be observable in the first-order spectra. For the polar A_1_ LO mode, the scattering cross-section depends not only on the deformation potential but also on the Fröhlich interaction within the material. Typically, the LO mode in ZnO exhibits weak intensity, as these two contributions tend to counteract each other.^70^ The E_2_ mode is associated with the motion of oxygen atoms and is sensitive to internal stress, characteristic of the hexagonal wurtzite structure in ZnO nanostructures.^71^

For the Cu hydroxide/oxide layer, Raman peaks corresponding to Cu_2_O were observed at the (LO) T_1_μ and T_2_g modes at 140 cm^–1^ and 484 cm^–1^, respectively. A dominant Raman peak for CuSO_4_ was also observed at 971 cm^–1^, which originates from the precursor material used,^72^ corresponding to the symmetric stretching vibration of the SO_4_^2–^ ion. The intensities of the ZnO peaks in these samples might be reduced due to the introduction of defects, impurities, or modifications to the crystal structure. The Raman peak intensity is observed to be related to the grain size: sharper and stronger Raman peaks are seen, which shift to higher wavenumbers as the grain size increases or decreases.^73,74^ Cu­(OH)_2_ peaks have been proven to be less prominent in Raman.^75^ According to group theory, the Raman spectrum of Cu_2_O should exhibit only one vibrational mode due to the 3-fold degenerate T_2_g symmetry. This mode is detected at 496 cm^–1^ in the Cu_2_O films. However, the experimental Raman spectrum can show additional peaks due to multiphoton processes (MPP). The T_2_g symmetry corresponds to the relative motion of the oxygen ions in the lattice. The T_1_u symmetry, on the other hand, is associated with the relative motion of the Cu and O lattices, involving the stretching and bending modes of asymmetric Cu–O and O–Cu–O bonds. The discrepancy between group theory predictions and the observed Raman spectra is a well-known effect, often attributed to nonstoichiometry in the samples.^76,77^ All of these observations were additions to the t-ZnO peaks already present.

For the Co layer, it was hard to distinguish between any of the desired peaks for cobalt hydroxide^78,79^ or oxides like CoO^80^ or Co_3_O_4_.^74,81^ This situation could be different for the Al layer, as Raman peaks for the Al layer were always observed predominantly at 242 cm^–1^, 306 cm^–1^, 323 cm^–1^, 395 cm^–1^, 430 cm^–1^, 539 cm^–1^, 568 cm^–1^, 708 cm^–1^, 810 cm^–1^, 890 cm^–1^, and 1012 cm^–1^.^82–84^ Interestingly, the peaks corresponding to t-ZnO either completely disappear or exhibit significantly reduced intensity upon aluminum (Al) coating. The peaks that shift to lower wavenumbers can be attributed to intrinsic defects in the host lattice of t-ZnO. These defects are activated as vibrating complexes due to the incorporation of Al.

The observed shifts may also indicate stress or strain induced by Al ions within the t-ZnO lattice. The number of frequencies associated with oxygen (O) modes in the 300–700 cm^–1^ range is notably less pronounced in the Al-coated t-ZnO compared to the uncoated t-ZnO microstructures. The E_2_ high mode for the Al-coated t-ZnO shifts slightly left, than the prominent peak for the uncoated t-ZnO lattice. This peak, which is close to the A_1_(LO) mode, has been assigned to surface optical modes as predicted theoretically, as well as to oxygen vacancies, ZnO defects, or a combination of the two. Other peaks previously observed in bare t-ZnO, such as those at higher wave numbers, were found to be absent in all Al-coated t-ZnO samples. Additionally, the peaks at 1100 and 1144 cm^–1^, which are typically associated with TO+LO combination modes at the M, L, and A, H points, also disappear in the Al-coated t-ZnO.^85^

Similarly, for the layer of Fe, Raman peaks were observed for the FeOOH phase at 526 cm^–1^, 665 cm^–1^ ^86^ and for Fe­(OH)_2_ at 251 cm^–1^.^87^ However, the peaks for FeOOH are observed to experience some right shifts from the literature. The wavelength that produces the strongest signal, 647 nm (corresponding to a photon energy of 1.92 eV), is associated with the transition from the ground state (6A_1_) to the excited ligand field state (4T_2_ (4G)).^86^ This transition leads to resonance, resulting in enhanced peak intensities. However, the Raman modes for Ni are similar to those for Co and remain indistinguishable as well. The Raman spectra have been depicted in Figure 6 for t-ZnO and shell metal hydroxide/oxides.

**Figure 6:**
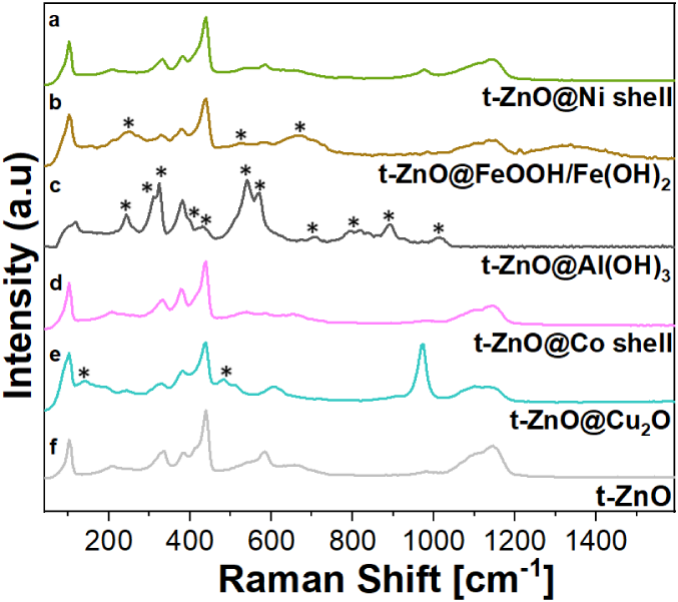
(a–e) Raman Spectra for the corresponding t-ZnO@metal hydroxide/oxide core–shell structures, and (f) bare t-ZnO.

### XRD

The t-ZnO structure was confirmed in all of the diffractograms in Figure 7. Specifically, these reflections match with the hexagonal wurtzite structure (Reference Code of Data Card as 98–001–1316). Thus, in most cases we could observe reflections at 2θ values of 31.83°, 34.50°, 36.33°, 47.65°, 56.72°, 63.01°, 66.53°, 68.11°, 69.25°, 72.75°, 77.15°, 81.60°, and 89.86° with corresponding (h k l) values as (0 1 0), (0 0 2), (0 1 1), (0 1 2), (1 1 0), (0 1 3), (0 2 0), (1 1 2), (0 2 1), (0 0 4), (0 2 2), (0 1 4), and (0 2 3), respectively. In certain patterns, some reflections were found to be more predominant than others. XRD reflections of the shell layers have a weak intensity due to their thickness in the nanometer range. For t-ZnO@Cu­(OH)_2_, the strongest reflection corresponding to Cu­(OH)_2_ (CAS Number: 20427–59–2; PDF No. 13–0420) is weakly observed at a 2θ value of 23.8°, corresponding to the (0 2 1) plane.^88^ Similarly, the highest intensity reflection was observed for Ni­(OH)_2_ around 10° for the (0 0 3) plane.^89^ However, no similar XRD features were observed for the Co and Fe shells formed around t-ZnO. The occasional shift toward a slightly lower 2θ angle (approximately 1 degree each) for Cu­(OH)_2_, Al_2_O_3_, and Ni­(OH)_2_ indicates an increase in lattice spacing. Owing to this limited information and also to study interactions at the interface, further studies were conducted at the DESY synchrotron PETRA III for a couple of these samples.

**Figure 7:**
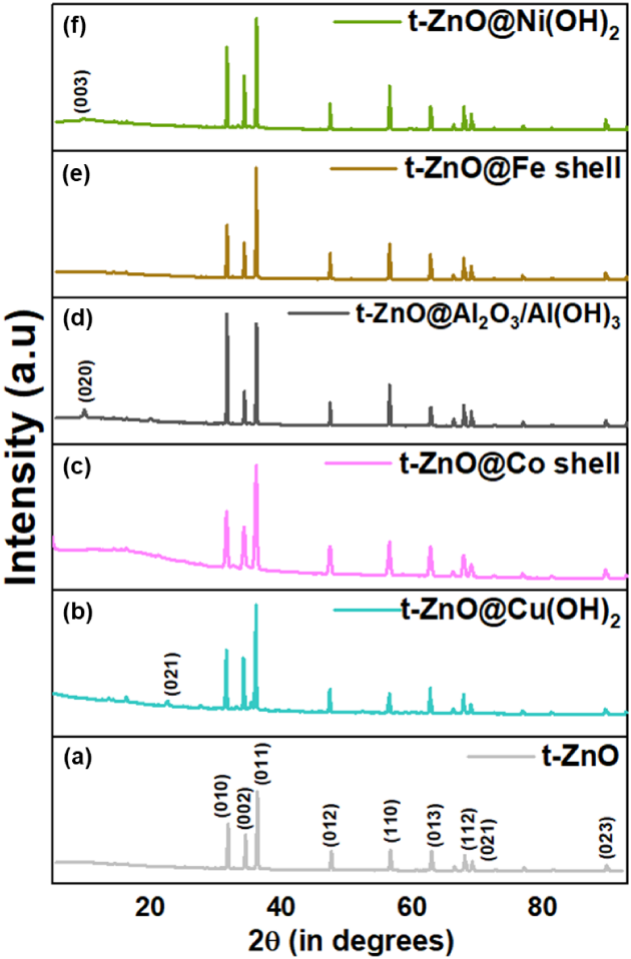
(a–e) XRD diffractograms of t-ZnO@metal hydroxides/oxides with t-ZnO as reference (in gray), and (f) t-ZnO.

The strong adhesion of the hydroxide/oxide shell to the t-ZnO core is attributed to the in situ surface conversion mechanism employed in this study. Unlike physical deposition processes, the shell forms directly through interfacial reactions at the t-ZnO surface, promoting chemical integration at the core–shell junction. The resulting structures remain intact after repeated washing, drying, and sonication steps, with no evidence of shell delamination. Notably, the shells also withstand the mechanically demanding cross-section preparation required for nano-XRD analysis, including polymer embedding, extrusion, and ultramicrotome cutting, without detachment. The preservation of intact core–shell cross sections under these harsh conditions provides strong evidence for a mechanically stable and chemically bonded interface.

### Interface Characterization by Nanodiffraction

Considering the raw diffraction images in Figure 7, it is apparent that the assumption of a powder sample is violated; therefore, not all diffraction peaks will be visible for the samples. Figure 8a–d shows the XRD and XRF maps of t-ZnO@CoO/Co­(OH)_2_ for distinct ZnO and Co_3_O_4_ peaks and Zn and Co, respectively. Supporting Figure 1 highlights the diffractograms of different locations of the maps for the t-ZnO@CoO/Co­(OH)_2_ and the t-ZnO@Al_2_O_3_/Al­(OH)_3_ sample, respectively.

**Figure 8:**
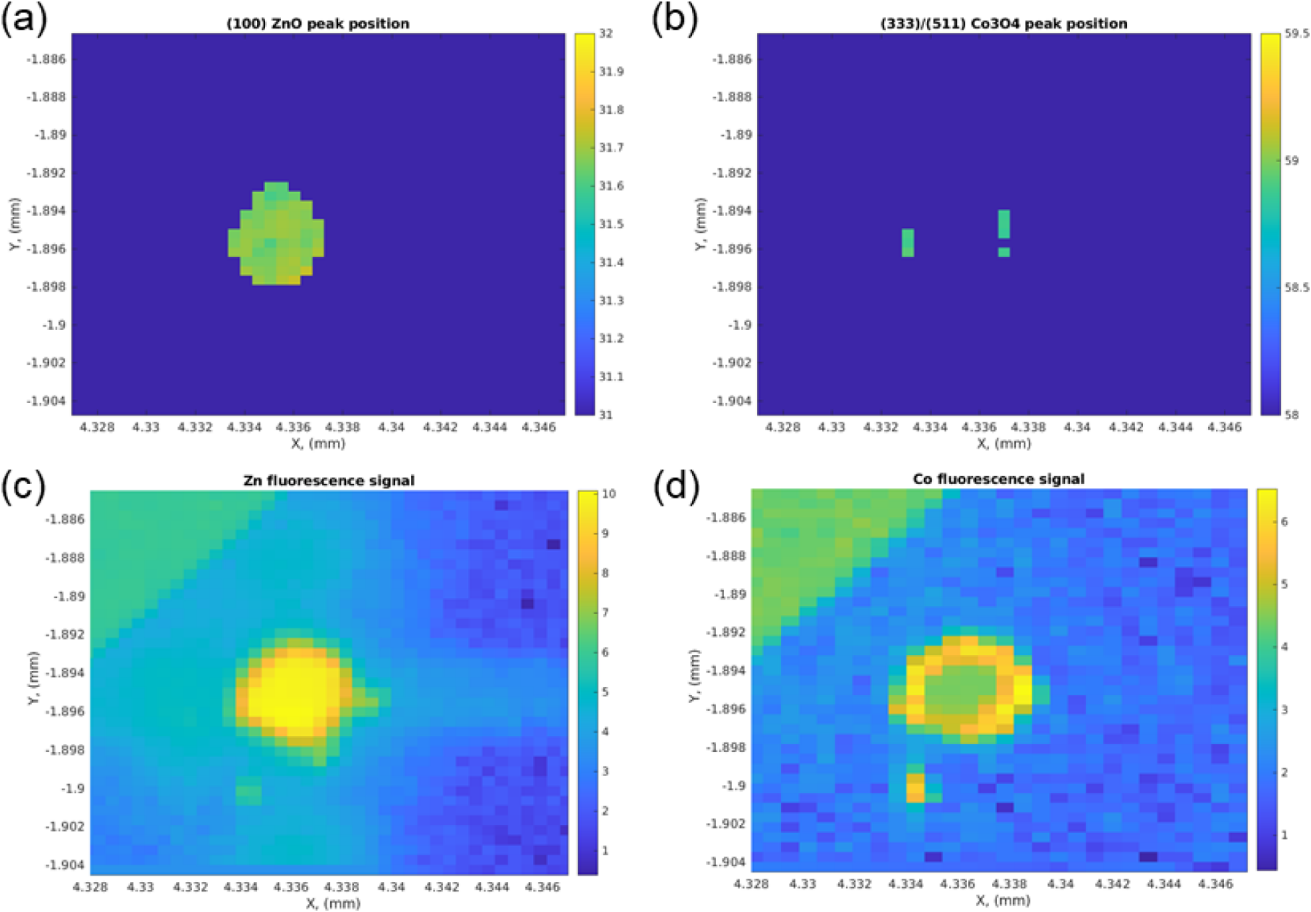
XRD maps of (a) the (1 0 0) ZnO peak, and (b) the presumed (3 3 3) or (5 1 1) Co_3_O_4_ peak, and corresponding XRF maps of (c) Zn and (d) Co.

The doped Co peaks appear to be distributed around the bulk t-ZnO. Co doping occurs at the t-ZnO@CoO/Co­(OH)_2_ interface in concentrations ranging from 3% to 5%.^90^ Since the powder average is not applicable, caution has to be taken with the interpretation of the data. The XRF data shows evidence for the presence of Co (Figure 1d). Due to the fact that we can no longer assume a powder average, it is possible that other regions also have crystals that cannot be detected, as they do not fulfill the Bragg condition. Alternatively, the remaining t-ZnO@CoO/Co­(OH)_2_ exhibits a highly amorphous nature. ^114^ Furthermore, the potential presence of Co_3_O_4_ can be inferred due to the appearance of a specific subset of peaks, some of which overlap with t-ZnO.^91^ In the t-ZnO@CoO/Co­(OH)_2_ sample, there is a distinct peak at approximately 33°, which could not be identified based on the current literature. Similarly, structures around 35.1–35.4° are observed, which could not be identified. This might be a sign for intermediate phases that have been formed.

The (0 0 2) peak at ∼18.16° is distinctly observable for Al­(OH)_3_, while faint traces of Al_2_O_3_ peaks can also be discerned. These spectral features are predominantly located at several edge pixels, corresponding to the periphery of bulk t-ZnO regions—specifically, the interface of t-ZnO@Al_2_O_3_/Al­(OH)_3._

The observed variation in shell thickness among different metal hydroxide/oxide systems can be rationalized by intrinsic differences in the metal ion chemistry, oxidation state stability, and reaction kinetics. For Cu, Co, and Ni, shell growth proceeds through comparatively slow hydrolysis and nucleation processes, likely involving diffusible intermediate species. This results in diffusion-limited growth and gradual shell thickening over long reaction times. In contrast, Al and Fe exhibit rapid hydrolysis and precipitation due to the high thermodynamic stability of their trivalent hydroxide/oxide phases, leading to reaction-limited growth and thicker shells formed within short reaction times. Consequently, the shell thickness variations reflect material-specific reaction pathways rather than differences in the t-ZnO template. For clarity and ease of comparison, the key compositional features, growth behavior, and typical shell thicknesses of the different metal hydroxide/oxide systems are summarized in Table 1.

**Table 1:** Summary of Shell Composition and Growth Behavior

Shell material	Dominantsurface phase	Reaction time	Growth behavior	Typical thickness
Cu	Cu(OH)_2_/CuO	1 h (RT)	Platelet-like, diffusion-limited	∼100–800 nm
Co	Co(OH)_2_/CoO	2 h (80 °C)	Platelet-like diffusion-limited	∼100–800 nm
Ni	Ni(OH)_2_/NiO	24 h (80 °C)	Slow, diffusion-limited	∼100–800 nm
Fe	FeOOH/Fe_2_O_3_	20 min(4 °C)	Mixed, reaction-limited	∼100–200 nm
Al	Al(OH)_3_/Al_2_O_3_	5 min (4 °C)	Rapid, reaction-limited	∼100–200 nm

## Conclusion

The synthesis of core@shell structures on t-ZnO represents a versatile and promising chemical synthetic approach for various applications, including sensing. The unique architecture, characterized by a core providing structural stability and a shell offering functional surface properties, enables enhanced functionality with a clear comparison. The template of t-ZnO is promising in its crystal structure and universal behavior in composite or core–shell structure formation. Its ability to integrate nanomaterials with tailored properties ensures a robust platform for advancing technologies. As the characterization becomes tricky with the thin nanolayer, therein lies the conductivity. The interface plays an important role by changing doping characteristics. Future work can also be done on such structures serving as the template for conversion to shell-based metal-organic frameworks such as ZIF-8, ZIF-71, and HKUST-1 for an integrated 3D conducting circuit for gas sensing.

## Supplementary Material



## Notes

Variation of shell-layer thickness with reaction time; supplementary SEM micrographs; EDX elemental maps for Cu, Al, Ni, and Fe coatings; additional XPS survey spectra and S 2p scans; supplementary nano-XRD diffractograms; and interface analysis (PDF)

## Abbreviations

(t-ZnO): Tetrapodal ZnO
(XRD): X-ray Diffraction
(SEM): Scanning Electron Microscopy
(XPS): X-ray Photoelectron Spectroscopy
(EDX): Energy Dispersive X-ray Analysis
(TEM): Transmission Electron Microscopy
(MOS): Metal Oxide Semiconductor
