# Supplementary material for: A Versatile Synthesis Approach and Interface Characterization of t‑ZnO@Metal Hydroxide/Oxide Heterostructures
Source: Cryst Growth Des. 2026 Feb 20;26(5):2038–51. doi: 10.1021/acs.cgd.5c01604 (PMC12965097; doi:10.1021/acs.cgd.5c01604)
Supplement: Supplementary file 1 [file cg5c01604_si_001.pdf]

A Versatile Synthesis Approach and Interface Characterization of t-ZnO@metal hydroxide/oxide Heterostructures

Barnika Chakraborty[1,2], Tim Tjardts[3], Berit Zeller-Plumhoff[4,5], Ulrich Schürmann[6], Anton Davydok[4], Dietmar Christian Florian Wieland[4], Haoyi Qiu[1], Alexander Reißmann[1], Nahomy Meling-Lizarde[3], Rajat Nagpal[1], Thomas Strunskus[3], Leonard Siebert.[1,7,], Rainer Adelung[1*]*

[1] Chair for Functional Nanomaterials, Department for Materials Science, Kiel University, Kaiserstr. 2, 24143, Kiel, Germany
[2] Micro- and Nanosystems (MNS), KU Leuven – University of Leuven, Kasteelpark Arenberg 10, 3001 Leuven, Belgium
[3] Chair for Multicomponent Materials, Department for Materials Science, Kiel University, Kaiserstr. 2, 24143 Kiel, Germany
[4] Institute of Metallic Biomaterials, Helmholtz-Zentrum Hereon, Max-Planck-Str. 1, 21502 Geesthacht, Germany
[5] Data-driven Analysis and Design of Materials, Faculty of Mechanical Engineering and Marine Technologies, University of Rostock, 18051 Rostock, Germany
[6] Synthesis and Real Structure Group, Department for Materials Science, Kiel University, Kaiserstr. 2, 24143 Kiel, Germany
[7] Centre for Surface Chemistry and Catalysis, KU Leuven – University of Leuven, Celestijnenlaan 200F, B-3001 Leuven, Belgium

Supporting Information-

Variation of shell-layer thickness with reaction time; supplementary SEM micrographs; EDX elemental maps for Cu, Al, Ni, and Fe coatings; additional XPS survey spectra and S 2p scans; supplementary nano-XRD diffractograms and interface analysis.

1. Variation of layer thickness of shell layer of metals

Further experiments with Copper under certain variation of reaction conditions proved the tunability of metal hydroxide/oxide layer thickness. Experimental analysis show 800nm-1000nm is the attainable range for the thickness of the metal layer of Cu for various metals of respective metal precursor (0.1 or 0.01 as was the standard maintained concentration in most cases), even when stored for longer durations, i.e. ~1 hour (standard reaction time for Copper). Thus an initial reduction of concentration to 0.01 M already helped in the reduction of the layer thickness from 800 nm further down to 400 nm. This was followed by the reduction of time to 30 mins, 5 mins and 5 secs. As far as Copper is concerned, it was quite evident that the reaction is much slower as compared to other metals such as iron or aluminium. In this case the nucleation happens at moderate speed and thus a hydroxide/oxide layer is not even seen to appear until 5 secs at room temperature (RT). Thereafter a growth of 150-250 nm is observed approximately after 5 secs of reaction progress. This increases to a range of 400-500 nm in 5 mins and to a larger extent of 800-900 nm range in 30 mins time. This condition will always

differ with the rate of layer growth of the metal derivative layers on the single crystal tetrapodal ZnO with specified oxygen vacancies. Thus the purpose was to elaborate on the fact that the tuning of layer thickness should be apparently possible for similarly spaced other metals too.

Some of the following SEM images would prove the uniformity of the outer layer growth throughout the material with the exception to the centre of the tetrapod and the face of the arm for all different metal derivative layers.

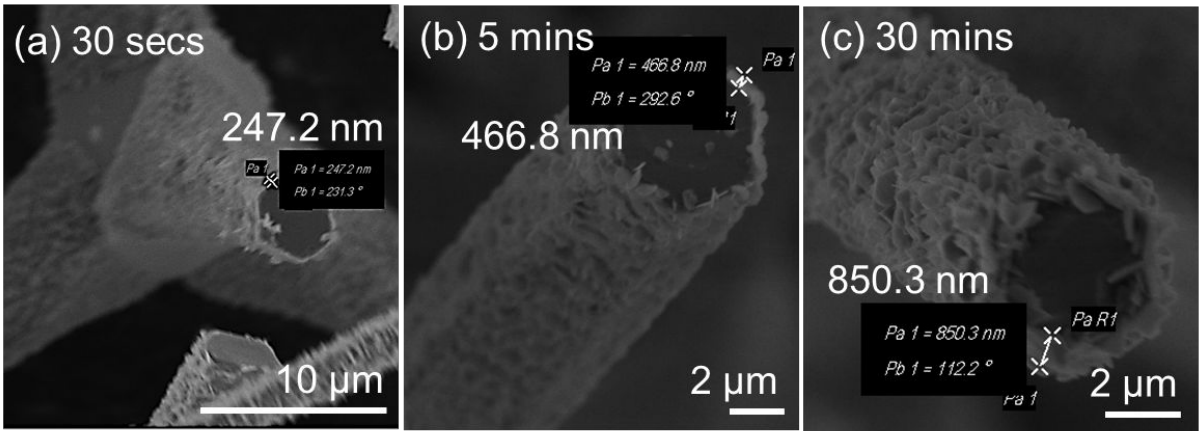

Figure1. Variation of layer thickness for shell layer to t-ZnO@$Cu(OH)_2$/CuO by variation of time from (a) 30 secs, (b) 5 mins, to (c) 30 mins

2. Enlisted maps for the metal hydroxide/oxide layers

Figure 2 continues to depict the EDX maps for all t-ZnO@metal hydroxide/oxide structures for Cu, Al, Ni, Fe in continuation to the Co, already established in the main manuscript. All these structures shows presence of Zn as core, corresponding metal (eg, Cu in bluish green, Al in red, Ni in green and Fe in dark yellow) as shell alongside eventual presence of O and C in all. Presence of O could be explained from the O in ZnO,hydroxides, oxides, etc. However, presence of C could be established from the adsorbed hydrocarbon mainly on the shell layers. S (mainly from the sulphate precursor) in presence in Cu, Al, Ni. Sometimes these samples were slightly sputtered in order to escape charging in the SEM. Na, occasionally recorded in the spectra is mainly due to energy overlap with Zn.

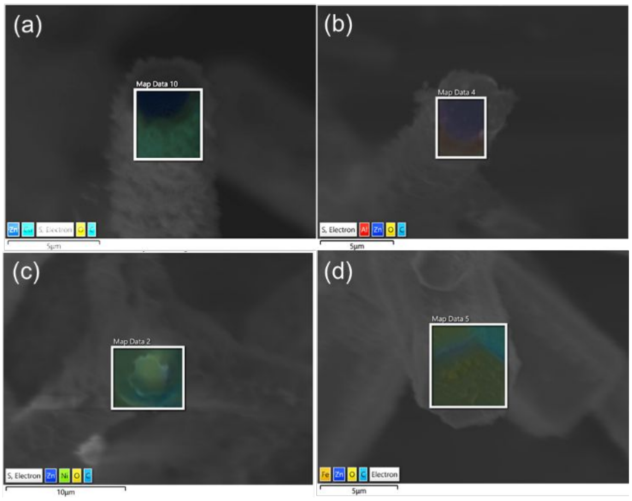

Figure 2a. Electron images for EDX Maps corresponding to (a) t-ZnO@CuO/$Cu(OH)_2$, (b) t-ZnO@Al_2O_3/$Al(OH)_3$, (c) t-ZnO@NiO/$Ni(OH)_2$, (d) t-ZnO@FeOOH, Fe_2O_3

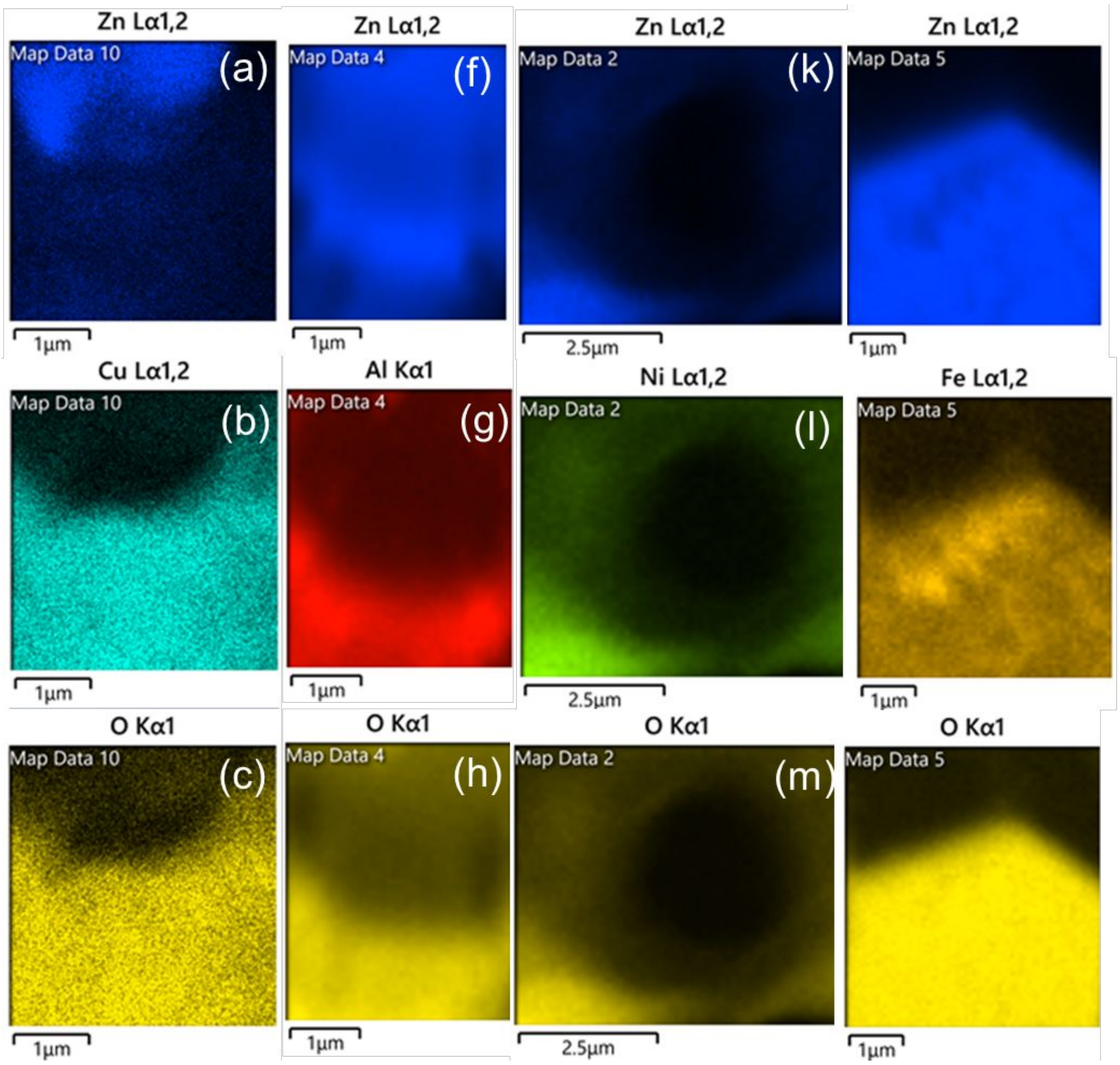

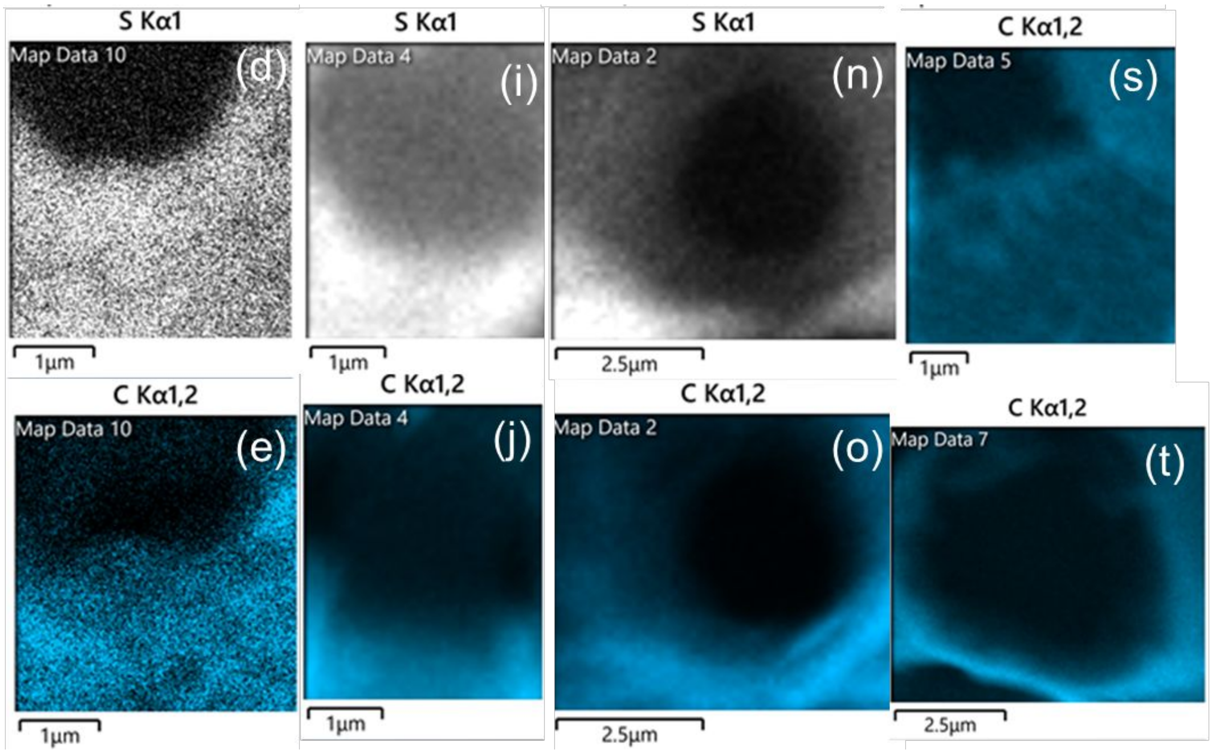

Figure 2b. Chemical composition mapping for Zn, corresponding shell metal (Cu, Al, Ni, Fe), O, S and C respectively for (a-e) t-ZnO@$Cu(OH)_2$/CuO, (f-j) t-ZnO@$Al(OH)_3$/Al_2O_3, (k-o) t-ZnO@ NiO/$Ni(OH)_2$, (p-s) t-ZnO@FeOOH/Fe_2O_3, (t) C from t-ZnO@CoO/$Co(OH)_2$

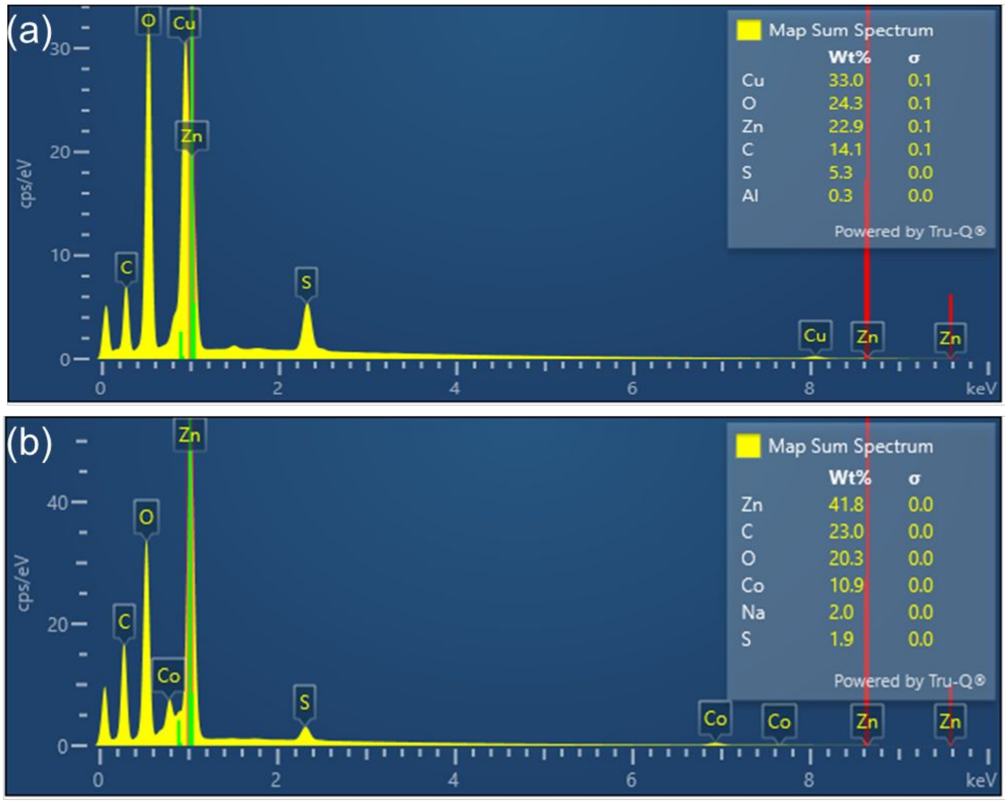

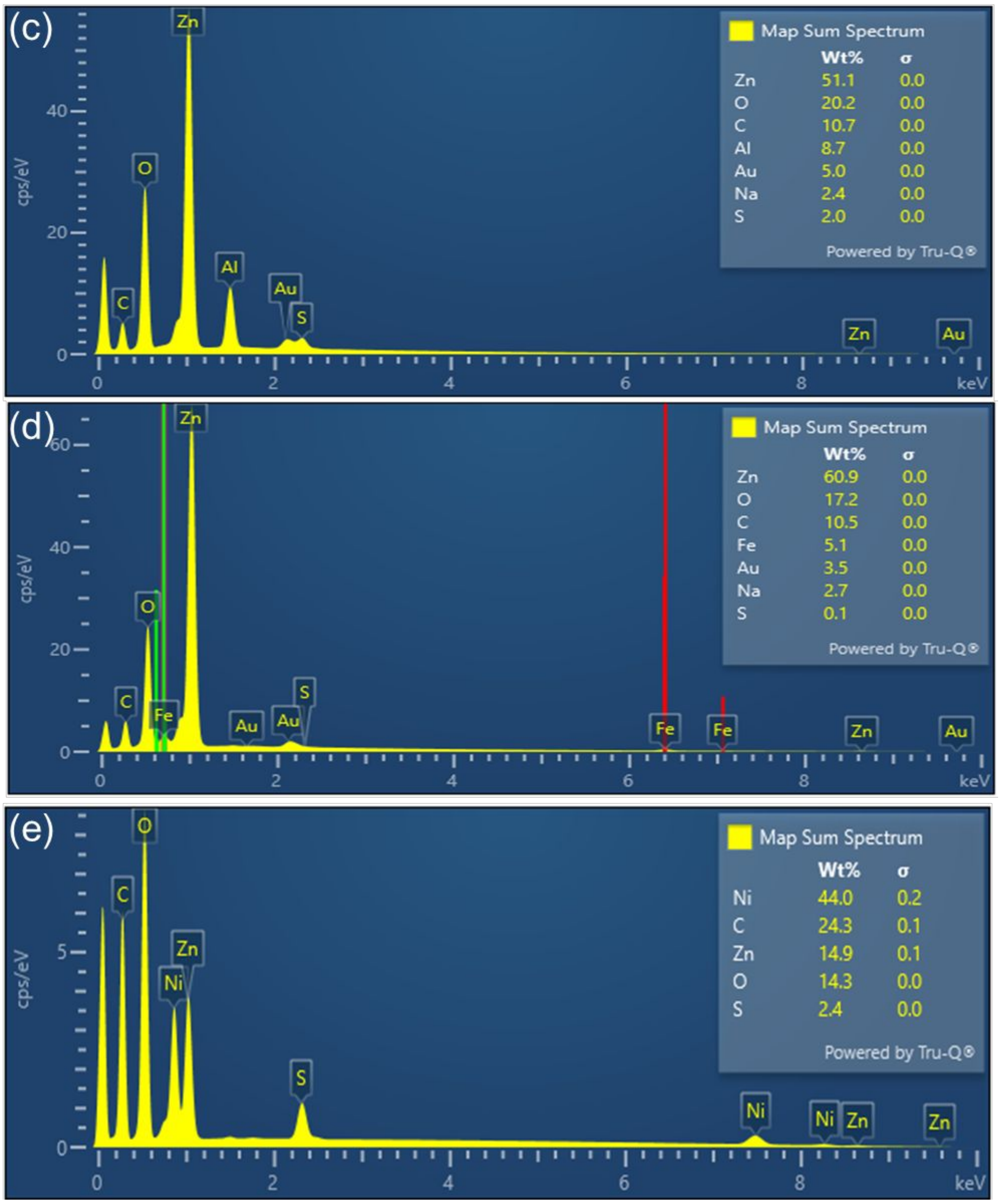

Figure 2c. Map Sum Spectrum for (a) t-ZnO@CuO/Cu$(OH)_2$, (b) t-ZnO@ CoO/Co$(OH)_2$ (c) Al_2O_3/Al$(OH)_3$, (d) t-ZnO@FeOOH/Fe_2O_3, (e) t-ZnO@NiO/Ni$(OH)_2$

3. **Additional Survey Scans and Sulphur Plots from XPS.**

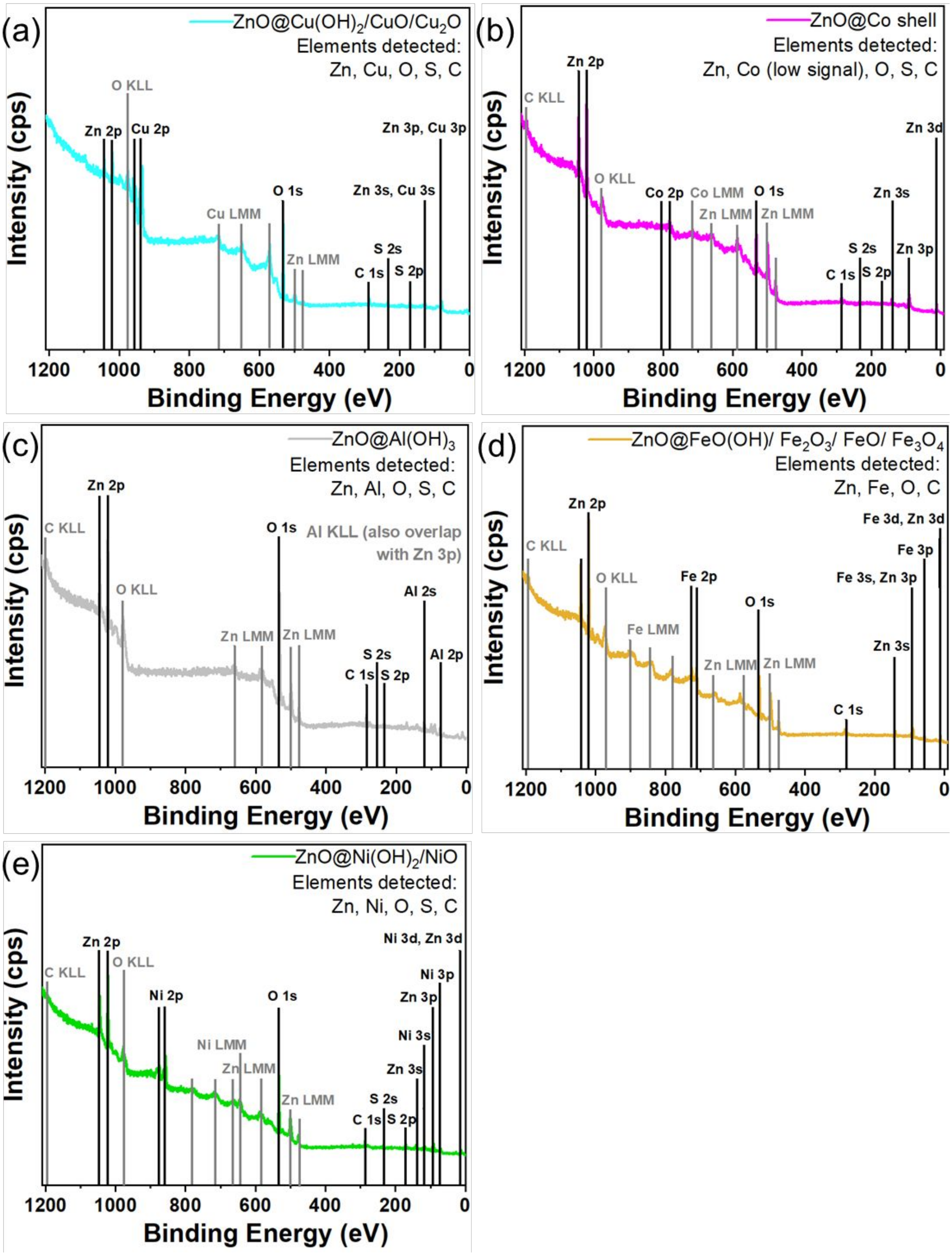

Figure 3a. XPS Survey scans for (a) t-ZnO@CuO/$Cu(OH)_2$, (b) t-ZnO@ CoO/$Co(OH)_2$ (c) Al_2O_3/$Al(OH)_3$, (d) t-ZnO@FeOOH/Fe_2O_3, (e) t-ZnO@NiO/$Ni(OH)_2$

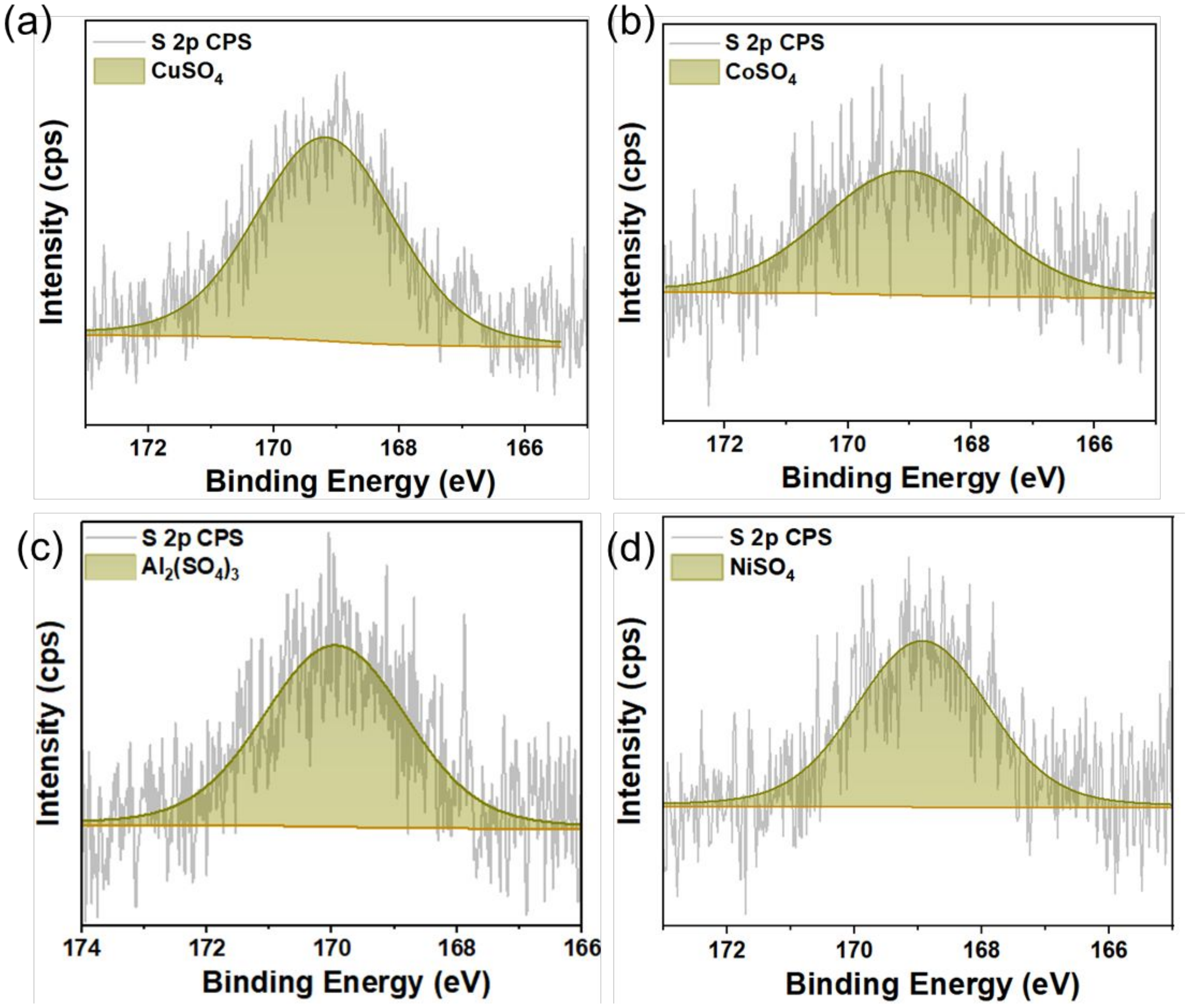

Figure 3b. S scans for (a) t-ZnO@CuO/Cu$(OH)_2$, (b) t-ZnO@ CoO/Co$(OH)_2$ (c) Al_2O_3/Al$(OH)_3$, (d) t-ZnO@NiO/Ni(OH)

4. Nanodiffraction studies for t-ZnO@$Al(OH)_3/Al_2O_3$ and t-ZnO@Co_3O_4

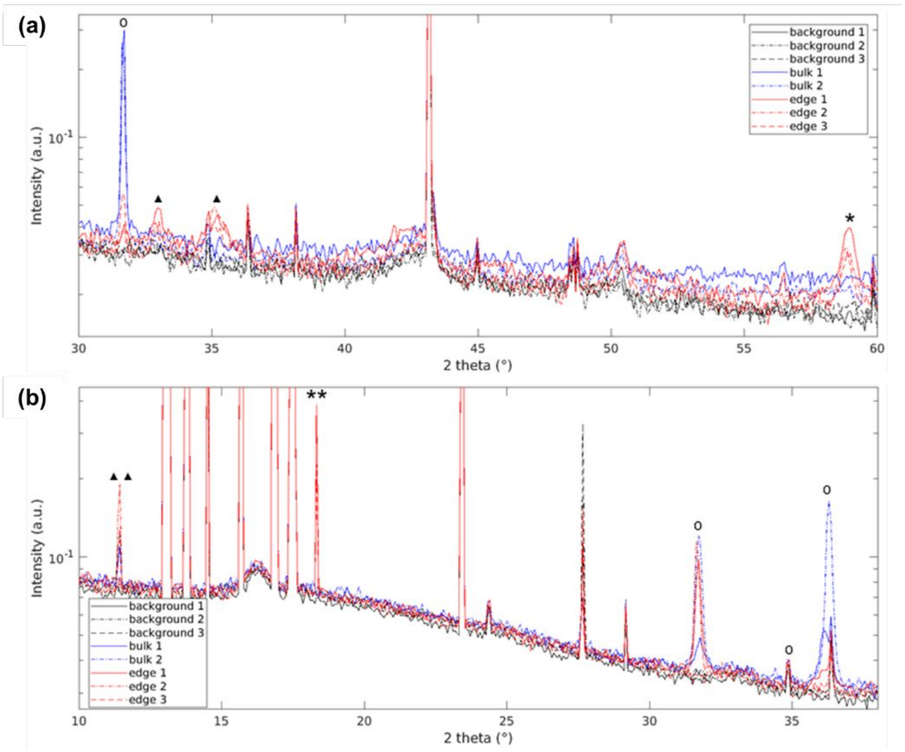

Figure4. Diffractogram for comparison of bulk to edge for (a) the same t-ZnO@Co sample and a (b) t-ZnO@$Al(OH)_3/Al_2O_3$ sample. o indicates ZnO peaks, * indicates Co_3O_4, ** indicates $Al(OH)_3$ and ▲ and ▲▲ indicates unidentified peaks for each sample, respectively. All other non-marked peaks appear across all pixels and are therefore presumed to relate to background scattering.

5. UV Sensing Pulsing of t-ZnO-metal hydroxide-oxide core-shell structures

The UV Sensing reproducibility pattern produces very fast recovery which could owe to the structural benefit and a high response with each pulse owing to a higher creation of oxygen vacancies with every UV pulse or a more cleansing effect on the surface.

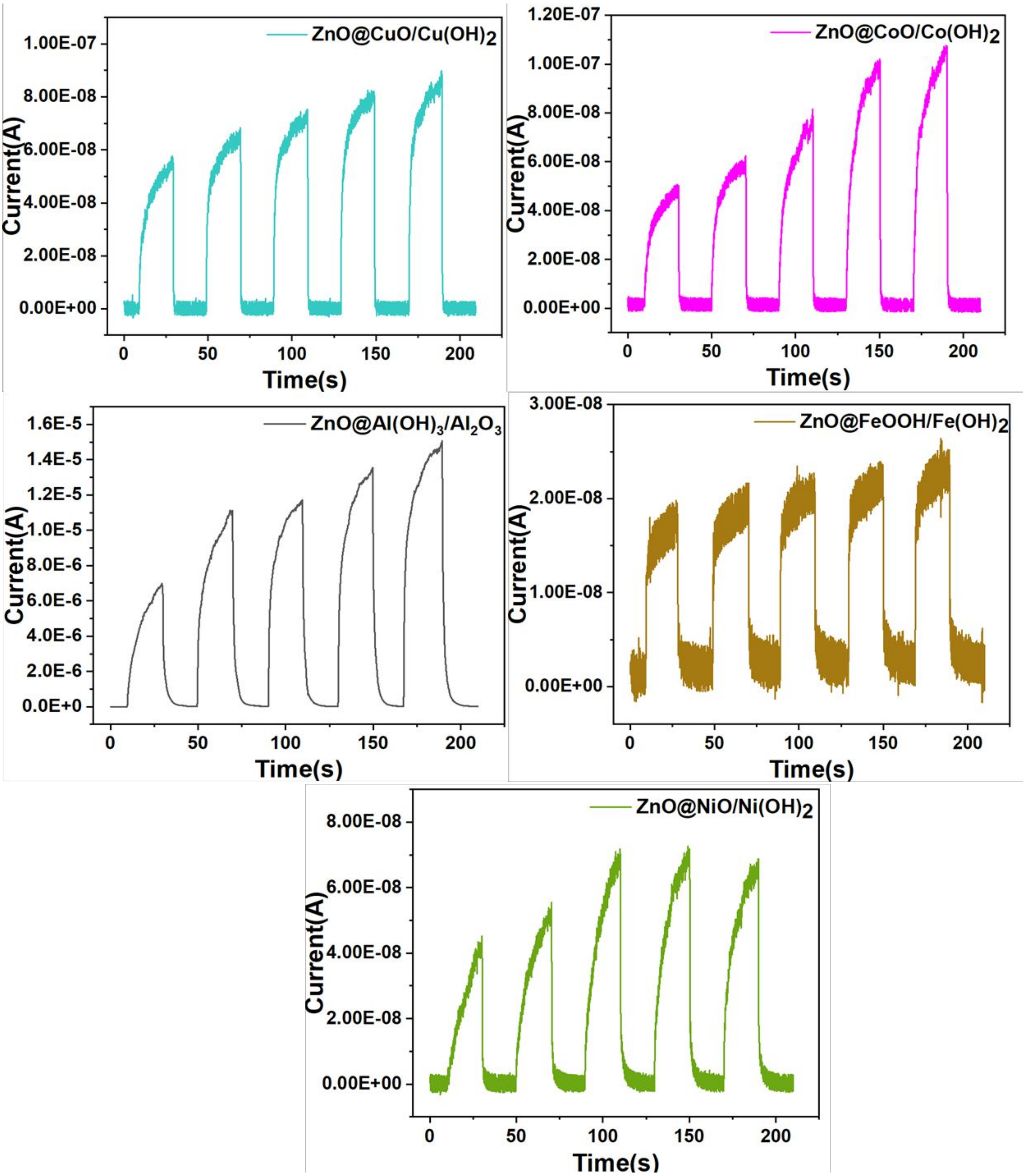

Figure 5. UV pulsing of t-ZnO- metal hydroxide/oxides
